# Editorial: Immunomodulatory strategies for managing viral infections: host immune responses and therapeutic targets

**DOI:** 10.3389/fcimb.2026.1795797

**Published:** 2026-02-12

**Authors:** Pratibha Gaur, Ekta Khattar, Ramendra Pati Pandey

**Affiliations:** 1 Department of Biotechnology and Microbiology, School of Science and Humanities, SRM University, Sonipat, Haryana, India; 2 Sunandan Divatia School of Science, SVKM’s Narsee Monjee Institute of Management Studies (NMIMS) (Deemed to be) University, Mumbai, India

**Keywords:** emerging viral infection, host immune response, management - healthcare, therapeutic targets, viral infection

## Introduction

Viral infections continue to pose a substantial global health burden, as underscored by recurrent outbreaks and pandemics caused by emerging and re-emerging viruses. While direct-acting antivirals and vaccines remain central to viral disease control, their effectiveness is often limited by viral mutation, immune escape, restricted availability, or variable host responses (Medzhitov, 2008; Vabret et al., 2020). Increasing evidence highlights that disease severity and clinical outcomes are frequently determined not only by viral replication but also by the nature, magnitude, and regulation of host immune responses (Iwasaki and Medzhitov, 2015; Blanco-Melo et al., 2020). Consequently, immunomodulatory strategies that fine-tune host immunity have emerged as a promising and complementary approach for managing viral infections.

The editorial summarizes the recent advances in understanding host immune responses to viral infections and immunological pathways that can be therapeutically targeted to improve disease outcomes. By bringing together original research articles and reviews, this collection explores the dual role of the immune system in antiviral defense and immunopathology, emphasizing the need for balanced immune modulation rather than indiscriminate immune suppression or activation Schultze and Aschenbrenner, 2021).

Several contributions in this collection focus on the innate immune system, which constitutes the first line of defense against viral pathogens. Innate immune recognition through pattern-recognition receptors such as Toll-like receptors and RIG-I-like receptors initiates antiviral interferon responses and inflammatory signaling cascades that are essential for viral control but may also drive immunopathology when dysregulated (Kawai and Akira, 2010; McNab et al., 2015).

The adaptive immune response is another central theme of this Research Topic. The included studies and reviews discuss virus-specific T cell and B cell responses, immune memory formation, and mechanisms of immune exhaustion during chronic or severe viral infections (Wherry and Kurachi, 2015; Crotty, 2019). Importantly, these articles underscore how impaired or maladaptive adaptive immunity can lead to viral persistence, reinfection, or heightened immunopathology, thereby identifying potential checkpoints for therapeutic intervention.

Research article by Corrao et al. describes the role of precision monitoring of immunological response in hospitalized COVID-19 patients. The authors suggest that immune patterns are measurable and can be tracked as immune trajectories which are beyond lab values obtained from single time point data. The study implicates that immune-pattern profiling could improve risk stratification and bedside monitoring in hospitalized patients, supporting better personalized decisions. It provides framework applicable for other viral and inflammatory diseases by combining innate and adaptive readouts for precision monitoring of patients (Lucas et al., 2020; Hadjadj et al., 2020).

Another research article by Yarlagadda et al. report role of nasal probiotics in enhancing mucosal immunity by reducing excessive cytokine storms without hindering antiviral interferons, and reporting strain-specific effects as bridges between current vaccines and broader prophylaxis (Belkaid and Hand, 2014).

Review article by Li et al. describe the innate immune role of IL-6 during influenza A virus (IAV) infection, highlighting its induction via pattern recognition receptors like TLRs and RLRs that activate NF-κB and other pathways in cell types including epithelial cells, macrophages, and dendritic cells (Tanaka et al., 2014). The authors suggest that targeting dysregulated IL-6 signaling offers therapeutic potential, such as with IL-6R antagonists like tocilizumab, to balance protective immunity against immunopathology in IAV and related viral infections (Rose-John, 2020).

Another minireview article by Patrick et al. provides insights into the protein domains of the C-VI TRIM subfamily (TRIM24, TRIM28, TRIM33), highlighting their RBCC motifs (RING, B-box, coiled-coil) that mediate various post translational modifications like ubiquitination, SUMOylation, and phosphorylation to regulate viral infections. These TRIM proteins exhibit dual roles: restricting viruses like HIV-1, HSV-1, and SARS-CoV-2 via proteasomal degradation of viral components or upregulating IFN responses, while some enhance their replication (e.g., TRIM33 degrading viperin). Therapeutic targeting of these domains offers potential for antiviral strategies by disrupting virus-TRIM interactions or amplifying restriction functions.

Collectively, the articles in this collection place immunomodulation within a broader translational and clinical context. They emphasize that successful management of viral infections requires a nuanced understanding of host–virus interactions, temporal dynamics of immune responses, and patient-specific immune landscapes. By integrating fundamental immunology with therapeutic perspectives, this collection aims to stimulate further research into rationally designed immunomodulatory interventions that can complement existing antiviral strategies (Vabret et al., 2020).

We hope that this collection will serve as a valuable resource for researchers and clinicians, fostering interdisciplinary dialogue and advancing the development of safe and effective host-targeted therapies for viral diseases.
